# A Virtual Cardiometabolic Health Program Among African Immigrants in the US: A Pilot Cluster-Randomized Clinical Trial

**DOI:** 10.1001/jamanetworkopen.2024.62559

**Published:** 2025-03-04

**Authors:** Oluwabunmi Ogungbe, Thomas Hinneh, Ruth-Alma N. Turkson-Ocran, Loretta Owusu, Baridosia Kumbe, Erin M. Spaulding, Serina Gbaba, Adeline Assani-Uva, Jasmine Mensah, Yvette Yeboah-Kordieh, Aminata Sinyan, Margaret Ampofo, Faith Oyedepo, Yvonne Commodore-Mensah

**Affiliations:** 1 Johns Hopkins University School of Nursing, Baltimore, Maryland; 2 Johns Hopkins Bloomberg School of Public Health, Baltimore, Maryland; 3 Beth Israel Deaconess Medical Center, Boston, Massachusetts; 4 Harvard Medical School, Boston, Massachusetts; 5 Office of Professional Practice and Nursing Education, Holy Cross Hospital, Silver Spring, Maryland; 6 Krieger School of Arts and Sciences, Johns Hopkins University, Baltimore, Maryland; 7 RoyalHouse Chapel International, Laurel, Maryland; 8 Winners Chapel International Inc, Bowie, Maryland

## Abstract

**Importance:**

Black persons, including immigrants, in the US disparately experience poor cardiometabolic health (CMH). Limited research on the effect of lifestyle interventions that improve CMH among African immigrant populations is available.

**Objective:**

To test the effectiveness of a culturally adapted, virtual lifestyle intervention on control of blood pressure (BP) and hemoglobin A_1c_ (HbA_1c_) levels among African immigrants with CMH risk factors.

**Design, Setting, and Participants:**

Afro-DPP (Diabetes Prevention Program), a pilot cluster-randomized clinical trial, evaluated the effectiveness of a multicomponent CMH intervention. The study took place in 2 churches with predominantly African immigrant congregations in the Baltimore–Washington, DC, metropolitan area from January 1, 2022, to July 31, 2023. Participants were adults aged 25 to 75 years with at least 2 CMH risk factors who self-identified as African immigrants and belonged to the participating churches. Analyses followed the intention-to-treat principle.

**Intervention:**

Participants received a 6-month culturally adapted lifestyle intervention based on the National DPP curriculum, delivered via virtual group sessions by a lifestyle coach of African origin. The delayed intervention began 6 months later with a follow-up time of 6 months. The intervention also included remote BP and weight monitoring.

**Main Outcome and Measures:**

Primary outcomes were changes in systolic and diastolic BP and HbA_1c_ levels from baseline to 6 months. Secondary outcomes included reduced body weight and body mass index (BMI; calculated as weight in kilograms divided by height in meters squared).

**Results:**

The analytic population included 60 participants (mean [SD] age, 50.6 [11.9] years; 40 [66.7%] women). In the first intervention group (n = 30), systolic BP decreased by 9.2 (95% CI, 2.5-15.9) mm Hg, diastolic BP by 6.1 (95% CI, 2.1-10.0) mm Hg, body weight by 4.9 (95% CI, 1.0-8.7) kg, and BMI by 1.1 (95% CI, 0.4-1.7) at 6 months. In the delayed intervention group (n = 30), systolic BP decreased by 11.4 (95% CI, 2.4-20.5) mm Hg, diastolic BP by 10.3 (95% CI, 5.4-15.2) mm Hg, and body weight by 3.3 (95% CI, 0.01-6.5) kg, while BMI increased by 0.3 (95% CI, −1.5 to 2.0).

**Conclusions and Relevance:**

Trial findings indicate that interventions incorporating cultural adaptation and virtual components could help address CMH disparities in this population.

**Trial Registration:**

ClincalTrials.gov Identifier NCT05144737

## Key Points

**Question:**

What is the effect of a culturally tailored, virtual, multicomponent cardiometabolic health intervention on blood pressure (BP) and glycemic control among African immigrants in the US with cardiometabolic risk factors?

**Findings:**

In this pilot cluster-randomized clinical trial that included 60 African immigrants, those in the first intervention group had systolic BP reduction of 9.2 mm Hg and diastolic BP reduction of 6.1 mm Hg; those in the delayed intervention group had systolic BP reduction of 11.4 mm Hg and diastolic BP reduction of 10.3 mm Hg at 6 months.

**Meaning:**

The findings suggest that adapting evidence-based lifestyle interventions by incorporating cultural elements and leveraging virtual platforms may be an effective strategy to improve cardiometabolic health among African immigrant populations.

## Teaser

This pilot cluster-randomized clinical trial evaluates the effectiveness of a virtual, culturally tailored lifestyle intervention with remote blood pressure monitoring on cardiometabolic health outcomes among African immigrants in the US.

## Introduction

Black people, including immigrants, in the US bear a disproportionate burden of cardiometabolic conditions, such as hypertension and type 2 diabetes.^1,2,3^ Despite effective interventions to improve cardiometabolic health (CMH), these populations continue to experience poorer outcomes compared with their non-Hispanic White counterparts.^4^ Among non-Hispanic Black adults, including immigrants, more than half (56%) have hypertension, a significantly higher prevalence than the 48% observed among non-Hispanic White adults.^5^ Similarly, 12.7% of non-Hispanic Black individuals have type 2 diabetes, compared with 11.0% of non-Hispanic White adults, with a substantial proportion remaining undiagnosed.^6^ This disparity in cardiometabolic burden results in higher mortality rates, increased disability, and substantial health care costs, totaling nearly $320 annually among Black people.^7^

African immigrants represent a rapidly growing and heterogeneous minoritized group in the US that is also affected by adverse CMH outcomes.^3^ Between 2000 and 2015, the Black immigrant population increased by 137%, reaching 2.1 million, with approximately 30% originating from Ghana and Nigeria.^8^ Persons of African heritage, including African immigrants, are often aggregated and categorized as “Black/African American,” overlooking the cultural diversity within this group and the implications of implementing culturally tailored interventions.^3,9^ A significant proportion of African immigrants experience a high burden of cardiometabolic disease risk.^2,10^ Factors contributing to these disparities include the cumulative impact of acculturation stress, limited access to culturally competent health care services, dietary transitions, and lifestyle changes associated with migration.^10,11,12^ Furthermore, many African immigrants already face a disproportionate cardiometabolic disease burden before migrating to the US.^13^

Evidence-based programs, such as the Centers for Disease Control and Prevention’s National Diabetes Prevention Program (DPP),^14^ have shown benefits in promoting weight loss through dietary and exercise strategies.^15,16^ However, participation among African immigrants and other ethnic minoritized populations remains low if the program is not tailored to incorporate relevant cultural beliefs, practices, and values.^17,18^ Prior studies have demonstrated the potential of collaborating with faith-based and religious organizations to deliver culturally adapted, sustainable lifestyle interventions for underserved communities.^19,20^

Integrating virtual platforms into health care delivery offers opportunities to facilitate self-monitoring of CMH indicators and disseminate health information. However, there is a paucity of rigorous research evaluating the effectiveness of culturally tailored, multicomponent virtual CMH programs designed specifically for African immigrant populations in the US. Addressing this gap is crucial to mitigate the disproportionate burden of cardiometabolic diseases in this rapidly growing population. This pilot clinical trial evaluated the effectiveness of a virtual, culturally tailored lifestyle intervention with remote blood pressure (BP) monitoring on CMH outcomes among African immigrants in Baltimore, Maryland, and Washington, DC metropolitan area. We hypothesized that a culturally tailored lifestyle intervention would improve BP and glycemic control among African immigrants with cardiometabolic risk factors.

## Methods

### Trial Design and Oversight

The Afro-DPP was a pilot cluster-randomized, parallel-group, unmasked clinical trial conducted from January 1, 2022, to July 31, 2023, to evaluate the effectiveness of a culturally tailored, multicomponent virtual CMH intervention among African immigrants with CMH risk factors. The institutional review board of The Johns Hopkins University approved the trial protocol and statistical analysis plan (Supplement 1). The study followed the Consolidated Standards of Reporting Trials (CONSORT) guidelines (eFigure 1 in Supplement 2), and an independent data and safety monitoring board provided oversight. All participants provided written informed consent.

### Participants

Eligible participants were adults who were born in an African country and subsequently immigrated to the US, aged 25 to 75 years, and had at least 2 of the following CMH risk factors: (1) body mass index (BMI; calculated as weight in kilograms divided by height in meters squared) of at least 25.0; (2) hemoglobin A_1c_ (HbA_1c_) level ranging from 5.7% to 6.5% (to convert to proportion of total hemoglobin, multiply by 0.01); and (3) systolic BP of at least 140 mm Hg or diastolic BP of at least 90 mm Hg. Smartphone ownership was a general inclusion criterion. Key exclusion criteria were cognitive impairment, severe chronic illness, inability to communicate in English, and nonmembership in the participating churches. We conducted targeted screenings with African churches, to improve enrollment of women in the study.

### Randomization and Masking

Two churches with predominantly African immigrant congregations in the Baltimore–Washington, DC, metropolitan area were recruited and served as cluster units. The churches were assigned to either the first intervention group or the delayed intervention group. Due to the interactive nature of the intervention, participants were informed of their group assignment, and study staff and data collectors were unmasked.

### Interventions

#### Lifestyle Intervention

The multicomponent intervention was adapted from the National DPP curriculum and focused on intensive lifestyle modification delivered by a certified lifestyle coach of African heritage. Educational materials were culturally tailored to incorporate African cultural values, practices, beliefs, and traditional dietary choices. The adapted DPP curriculum is described briefly in the trial protocol in Supplement 1.

#### First Intervention Group

Participants randomized to the first intervention group immediately began the 6-month adapted DPP lifestyle intervention, which included weekly 60-minute virtual group sessions via videoconferencing. The sessions covered topics such as stress management, healthy nutritional choices, physical activity, sleep considerations, and strategies to reduce cardiometabolic risk. Participants received a copy of the curriculum to follow along during the sessions.

#### Delayed Intervention Group

Participants in the delayed intervention group received no lifestyle intervention during the first 6 months but underwent the same assessments as the first intervention group. After 6 months, they received the identical adapted DPP lifestyle intervention as the first group, including remote monitoring.

### Remote Monitoring

All participants received a Bluetooth-enabled BP monitor (BP7250; Omron) and digital weight scale (BCM-500; Omron) at enrollment to enable remote monitoring of BP and body composition measures. The delayed intervention group served as an attention control with device monitoring only during the first 6 months. Participants were trained on using these devices for daily home measurements, which synchronized automatically to a smartphone app (Sphygmo) and a cloud-based platform accessible to the research team. This allowed for prompt feedback and reminders to enable participants to adhere to their prescribed medications and contact their clinicians for review of their treatment plan based on participants’ transmitted BP values. Details are included in the protocol (Supplement 1).

### Outcome Measures

Consistent with the trial registration, the primary outcomes were (1) changes in clinic-measured systolic and diastolic BP from baseline to 6 months using a validated automated device (HEM-9210T [Omron] and the Omron 10 series) by trained research staff following standardized procedures and (2) changes in HbA_1c_ levels (measured using the A1CNow+ point of care system; PTS Diagnostics). Prespecified secondary outcomes included changes in body weight and BMI during the same period.

### Assessments and Follow-Up

Participants attended in-person study visits at their respective churches for assessments at baseline, 1 month, 3 months, and 6 months. During these visits, trained staff followed standardized protocols to systematically measure BP, HbA_1c_ levels, body weight, and BMI. At each visit, trained staff shared and discussed the measurement results with each participant at every site.

### Statistical Analysis

All analyses followed the intention-to-treat principle. This hypothesis-generating pilot study was designed to provide preliminary data and estimate effect sizes to inform a larger-scale trial. Analyses were performed using Stata, version 16 (StataCorp LLC). Baseline characteristics were summarized using descriptive statistics and compared between groups using χ^2^ tests for categorical variables and 2-sample *t* tests for continuous variables. Complete data were available for age, educational level, employment status, and baseline physical measurements (30 per group). Other measures had varying numbers of participants with available data, as detailed in Table 1. Changes in primary (systolic and diastolic BP and HbA_1c_ levels) and secondary (body weight and BMI) outcomes over time were examined using linear mixed-effects models with fixed effects for time (baseline and 1, 3, and 6 months), intervention group (first or delayed), and their interaction. The linear mixed-effects models handled missing data under the missing at random assumption through maximum likelihood estimation. Random intercepts were included to account for clustering within churches. We estimated mean changes from baseline adjusting for age, sex, educational level, BP medication, and current alcohol use with 95% CIs. Effect sizes were calculated using Cohen *d* for continuous outcomes and number needed to treat for binary outcomes (eFigures 1-3 in Supplement 2). Visual representations of the model-based means over time were created using line plots with separate lines for each study group. A 2-sided *P* < .05 was considered statistically significant for all tests. No adjustments for multiple comparisons were made in this exploratory pilot study.

**Table 1.** Baseline Characteristics of the Afro-DPP (Diabetes Prevention Program) Trial

Variable	Intervention group	
	First (n = 30)	Delayed (n = 30)
Demographic characteristics		
Age, mean (SD), y	51.1 (9.5)	49.7 (15.3)
Sex, No./total No. (%)		
Female	24/30 (80.0)	16/30 (53.3)
Male	6/30 (20.0)	14/30 (46.7)
Length of stay in the US, mean (SD), y	9.4 (1.4)	20.9 (3.5)
Educational level above bachelor’s degree, No./total No. (%)	17/30 (56.7)	23/30 (76.7)
Employed, No./total No. (%)	20 (66.7)	14 (46.7)
Married, No./total No. (%)	18/21 (85.7)	12/20 (60.0)
Income <$50 000, No./total No. (%)^a^	8/19 (42.1)	2/22 (9.1)
Insured, No./total No. (%)	18/30 (60.0)	12/30 (40.0)
Health behaviors		
Alcohol use, No./total No. (%)	1/19 (5.3)	3/19 (15.8)
Diet, mean (SD), No. of servings		
Fruit	1.6 (0.9)	2.0 (2.5)
Vegetable	1.5 (0.2)	1.6 (0.3)
Physical activity, mean (SD), minutes		
Moderate	20.8 (2.8)	22.9 (3.5)
Vigorous	21.7 (2.5)	26.4 (4.8)
Medical history, No./total No. (%)		
History of hypertension	16/25 (64.0)	11/28 (39.3)
Blood pressure medication use	12/27 (44.4)	25/29 (86.2)
History of prediabetes	6/26 (23.1)	3/27 (11.1)
History of hypercholesterolemia	6/25 (24.0)	5/28 (17.9)
Baseline physical measurements		
Blood pressure, mean (SD), mm Hg		
Systolic	144.4 (24.6)	148.3 (14.6)
Diastolic	94.6 (15.7)	97.5 (14.3)
Hemoglobin A_1c_ level, mean (SD), %	5.6 (0.1)	5.3 (0.1)
Body weight, mean (SD), kg	220.1 (6.9)	192.4 (24.5)
BMI, mean (SD)	35.8 (6.4)	31.2 (4.6)
BMI categories, No. (%)		
Normal	0	1 (3.3)
Overweight	7 (23.2)	12 (40.0)
Obesity	23 (76.7)	17 (56.7)
Body fat percentage, mean (SD)	44.6 (9.4)	34.7 (9.8)
Country of origin, No.		
Ghana	27	2
Nigeria	1	17
Cameroun	0	4
Sierra Leone	0	3

Abbreviation: BMI, body mass index (calculated as weight in kilograms divided by height in meters squared).

SI conversion factor: To convert hemoglobin A_1c_ to proportion of total hemoglobin, multiply by 0.01.

^a^ Annual household income was dichotomized at $50 000 based on the median income from the sample.

## Results

### Participant Enrollment and Sociodemographic and Clinical Characteristics

**Figure 1.**
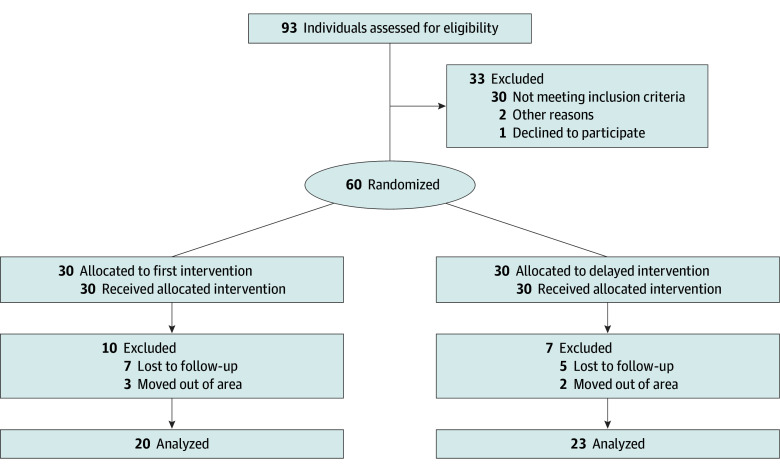
Flow of Participants Through a Virtual Cardiometabolic Health Program to Improve Blood Pressure and Glycemic Control Among African Immigrants in the US

A total of 60 participants were enrolled and randomized, 30 each to the first intervention and the delayed intervention groups (Figure 1). The mean (SD) participant age was 50.6 (11.9) years; 40 participants (66.7%) were female and 20 (33.3%) were male. The mean (SD) length of stay in the US was 9.4 (1.4) years in the first intervention group and 20.9 (3.5) years in the delayed intervention group. The groups were well-balanced on most baseline characteristics (Table 1); the first intervention group had a higher proportion of participants with income less than $50 000 compared with the delayed intervention group (8 of 19 [42.1%] vs 2 of 22 [9.1%]). The first intervention group had a higher baseline mean (SD) HbA_1c_ level (5.6% [0.1%] vs 5.3% [0.1%]), body weight (220.1 [6.9] vs 192.4 [24.5] kg), BMI (35.8 [6.4] vs 31.2 [4.6]), and body fat percentage (44.6% [9.4%] vs 34.7% [9.8%]) compared with the delayed intervention group. Both groups had similar mean (SD) baseline systolic BP (144.4 [24.6] vs 148.3 [14.6] mm Hg) and diastolic BP (94.6 [15.7] vs 97.5 [14.3] mm Hg). The self-reported prevalences of hypertension, prediabetes, and hypercholesterolemia were also similar between the groups. The retention rate was 45 patients (75.0%) at 3 months and 43 patients (71.7%) at 6 months (Figure 1).

### Primary Outcomes

#### Systolic and Diastolic BP

**Figure 2.**
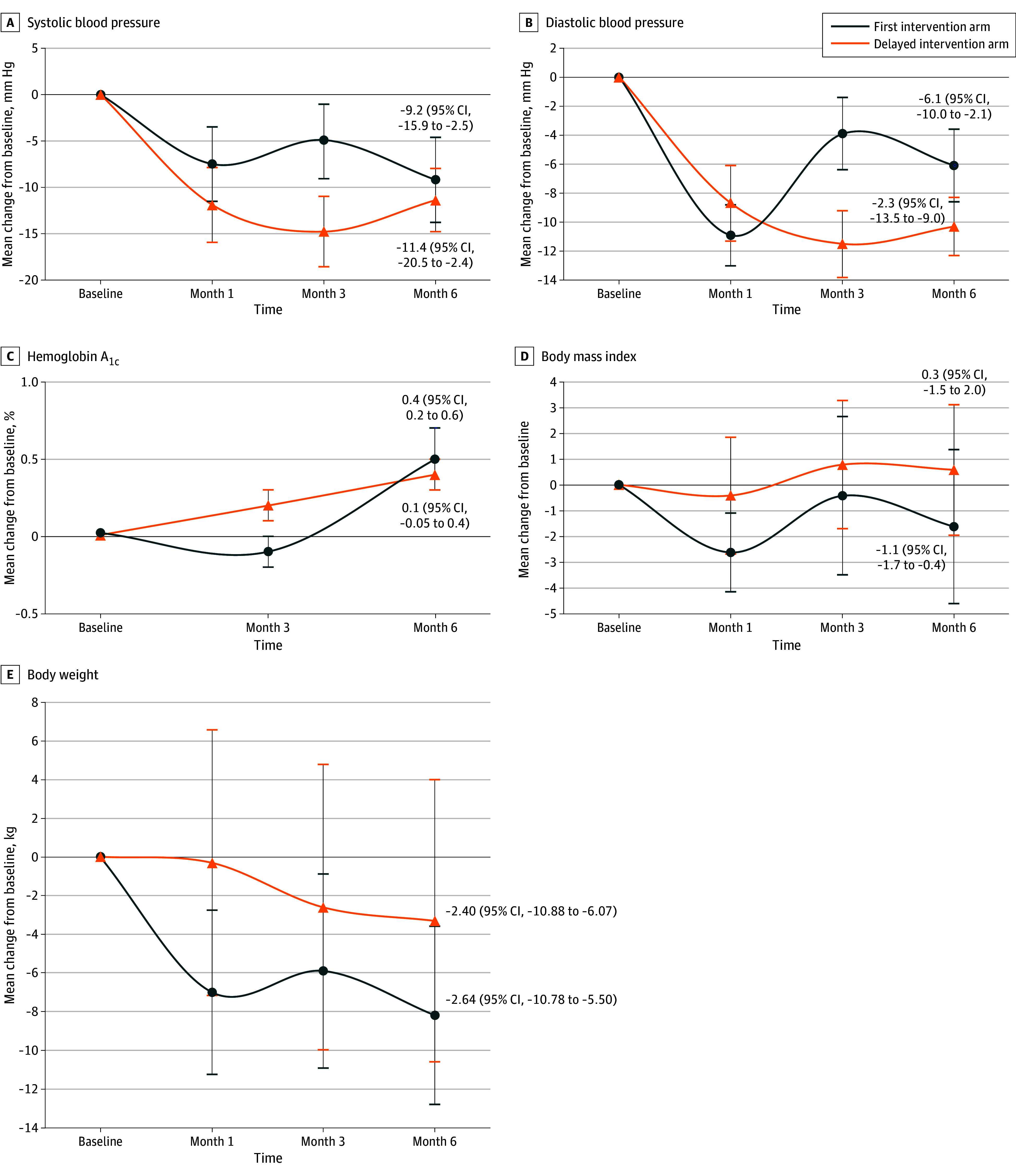
Changes in Primary and Secondary Outcomes Among African Immigrants Includes 30 participants per study arm in the Afro-DPP (Diabetes Prevention Program), a virtual cardiometabolic health intervention. Body mass index is calculated as the weight in kilograms divided by the height in meters squared.

**Figure 3.**
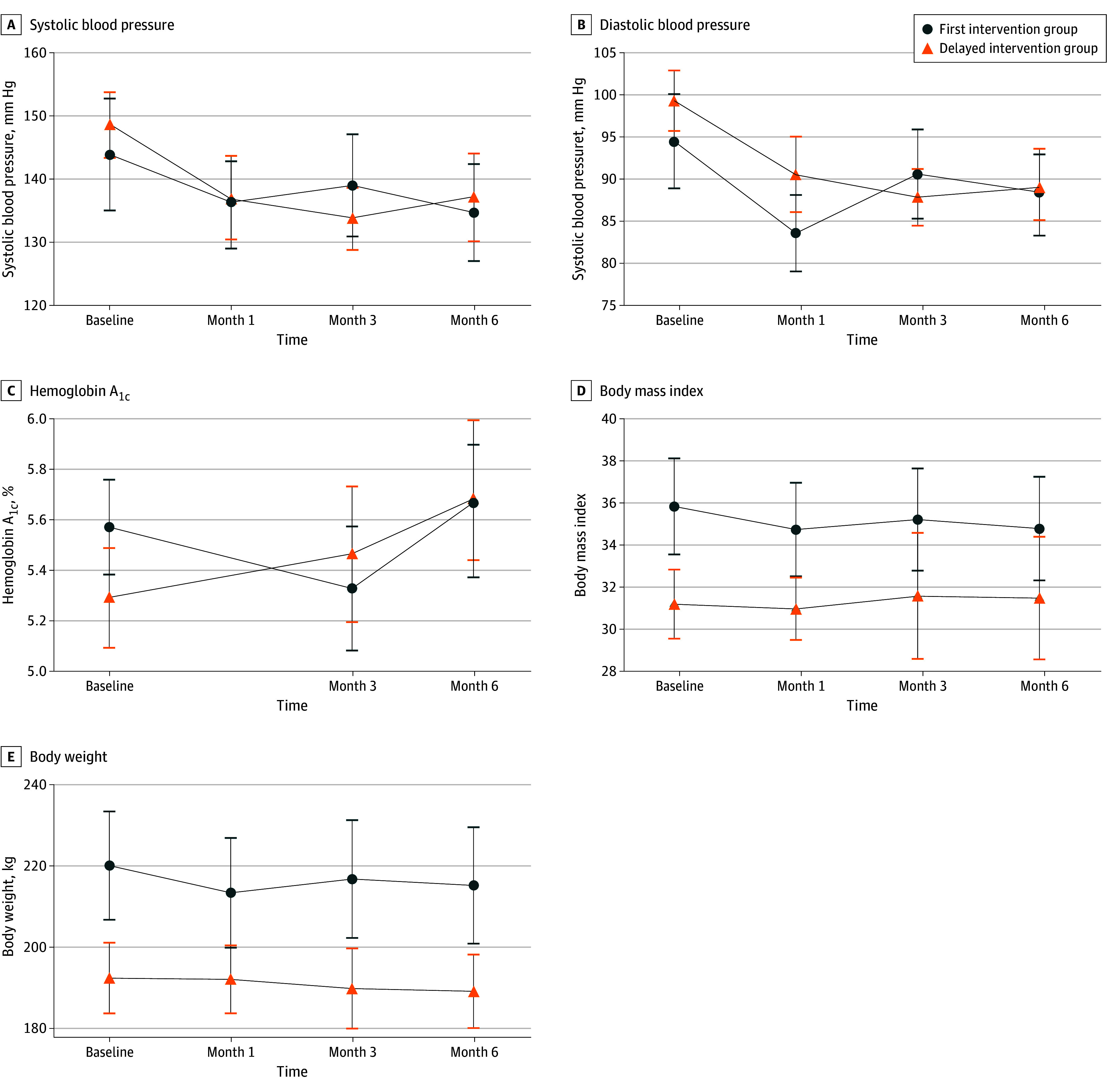
Mean Changes Over Time in Key Outcomes Among African Immigrants Includes 30 participants per study arm in the Afro-DPP (Diabetes Prevention Program), a virtual cardiometabolic health intervention. Body mass index is calculated as the weight in kilograms divided by the height in meters squared.

For systolic BP, there was a significant interaction between the study group and time (*P* = .001), indicating that the change in systolic BP over time differed between the 2 groups. The first intervention group had a mean reduction in systolic BP of 9.2 (95% CI, 2.5-15.9) mm Hg (*P* = .01) at 6 months, while the delayed intervention group had a mean reduction of 11.4 (95% CI, 2.4-20.5) mm Hg (*P* = .01). The difference between groups at 6 months was not statistically significant (−2.3 mm Hg; 95% CI, −13.5 to 9.0 mm Hg; *P* = .69) (Table 2, Figure 2A, and Figure 3A).

**Table 2.** Change in Primary and Secondary Outcomes First and Delayed Intervention Groups

Outcome^a^	Intervention group								Absolute difference^c^	*P* value^d^
	First				Delayed					
	Baseline	Final	Difference^b^	*P* value	Baseline	Final	Difference^b^	*P* value		
**Month 1**										
Primary outcome										
Systolic BP, mm Hg	143.9 (135.0 to 152.7)	136.3 (129.0 to 143.7)	−7.5 (−15.5 to 0.4)	.06	148.5 (143.3 to 153.7)	136.6 (130.4 to 142.8)	−11.9 (−19.8 to −4.0)	.003	−2.0 (−717.9 to 13.8)	.44
Diastolic BP, mm Hg	94.5 (88.9 to 100.1)	83.6 (79.0 to 88.1)	−10.9 (−16.0 to −5.8)	<.001	99.3 (95.7 to 102.9)	90.5 (86.1 to 95.0)	−8.7 (−12.9, −4.6)	<.001	2.2 (−4.4 to 8.8)	.52
Secondary outcomes										
Body weight, kg	220.1 (206.8 to 233.4)	213.4 (200.0 to 226.9)	−6.7 (−9.2 to −4.2)	<.001	192.4 (183.7 to 201.1)	192.1 (183.7 to 200.4)	−0.3 (−3.3 to 2.7)	.83	6.4 (2.5 to 10.2)	.001
BMI	35.8 (33.6 to 38.1)	34.7 (32.5 to 37.0)	−1.1 (−1.6 to −0.6)	<.001	31.2 (29.6 to 32.8)	31.0 (29.5 to 32.5)	−0.2 (−0.9 to 0.4)	.49	0.9 (0.1 to 1.7)	.03
**Month 3**										
Primary outcome										
Systolic BP, mm Hg	143.9 (146.3 to 150.3)	139.0 (130.9 to 147.1)	−4.9 (−12.2 to 2.5)	.19	148.5 (143.3 to 153.7)	133.7 (128.8 to 138.6)	−14.8 (−22.3 to −7.4)	<.001	−7.9 (−22.1 to 6.4)	.06
Diastolic BP, mm Hg	94.5 (88.9 to 100.1)	90.6 (85.3 to 95.9)	−3.9 (−8.4 to 0.6)	.09	99.3 (95.7 to 102.9)	87.8 (84.5 to 91.2)	−11.5 (−16.3 to −6.6)	<.001	−7.6 (−14.2 to −1.0)	.02
Hemoglobin A_1c_ level, %	5.6 (5.4 to 5.8)	5.3 (5.1 to 5.6)	−0.3 (−0.4 to −0.1)	.01	5.3 (5.1 to 5.5)	5.5 (5.2 to 5.7)	0.2 (−0.05 to 0.2)	.19	0.4 (0.1 to 0.7)	.01
Secondary outcomes										
Body weight, kg	220.1 (206.8 to 233.4)	216.8 (202.3 to 231.3)	−3.3 (−7.1 to 0.5)	.09	192.4 (183.7 to 201.1)	190.0 (180.0 to 199.7)	−2.6 (−6.6 to 1.5)	.21	0.7 (−4.8 to 6.3)	.80
BMI	35.8 (33.6 to 38.1)	35.2 (32.8 to 37.6)	−0.6 (−1.2 to 0.002)	.051	31.2 (29.6 to 32.8)	31.6 (28.6 to 34.6)	0.4 (−1.4 to 2.2)	.67	1.0 (−0.9 to 2.9)	.30
**Month 6**										
Primary outcome										
Systolic BP, mm Hg	143.9 (146.3 to 150.3)	134.7 (127.0 to 142.4)	−9.2 (−15.9 to −2.5)	.01	148.5 (143.3 to 153.7)	137.1 (130.1 to 144.0)	−11.4 (−20.5 to −2.4)	.01	−2.3 (−13.5 to 9.0)	.69
Diastolic BP, mm Hg	94.5 (88.9 to 100.1)	88.4 (83.3 to 93.6)	−6.1 (−10.0 to −2.1)	.003	99.3 (95.7 to 102.9)	89.0 (85.1 to 92.9)	−10.3 (−15.2 to −5.4)	<.001	−4.2 (−10.5 to 2.1)	.19
Hemoglobin A_1c_ level, %	5.6 (5.4 to 5.8)	5.7 (5.4 to 5.9)	0.1 (−0.05 to 0.4)	.18	5.3 (5.1 to 5.5)	5.7 (5.4 to 6.0)	0.4 (0.2 to 0.6)	<.001	0.3 (0.03 to 0.6)	.03
Secondary outcomes										
Body weight, kg	220.1 (206.8 to 233.4)	215.2 (200.9 to 229.5)	−4.9 (−8.7 to −1.0)	.01	192.4 (183.7 to 201.1)	189.1 (180.1 to 198.2)	−3.3 (−6.5 to −0.01)	.050	1.6 (−3.4 to 6.7)	.63
BMI	35.8 (33.0 to 38.1)	34.8 (32.2 to 37.2)	−1.1 (−1.7 to −0.4)	.003	31.2 (29.6 to 32.8)	31.5 (28.5 to 34.4)	0.3 (−1.5 to 2.0)	.75	1.3 (−0.5 to 3.2)	.16

Abbreviations: BMI, body mass index (calculated as the weight in kilograms divided by the height in meters squared); BP, blood pressure.

SI conversion factor: To convert hemoglobin A_1c_ to proportion of total hemoglobin, multiply by 0.01.

^a^ Sample size and model-adjusted means and 95% CIs are reported for outcomes, using linear mixed-effects models with fixed effects for time (baseline and 1, 3, and 6 months), intervention group (first or delayed), and their interaction. Random intercepts were included to account for clustering within churches.

^b^ Represents the difference between the change from baseline to the specified time point in the first intervention group and the delayed intervention group. *P* values for the absolute difference are reported.

^c^ Represents the difference between the change from baseline to the specified time point in the first intervention group and the change from baseline to the specified time point in the delayed intervention group. *P* values for the absolute difference are reported.

^d^ Calculated as the difference in differences between baseline and final values within each group are reported.

Similarly, for diastolic BP, there was a significant interaction between study group and time (*P* < .001). The first intervention group had a mean reduction in diastolic BP of 6.1 (95% CI, 2.1-10.0) mm Hg (*P* = .003) at 6 months, while the delayed intervention group had a mean reduction of 10.3 (95% CI, 5.4-15.2) mm Hg (*P* < .001). The difference between groups at 6 months was not statistically significant (−4.2 mm Hg; 95% CI, −10.5 to 2.1 mm Hg; *P* = .19) (Table 2, Figure 2B, and Figure 3B).

#### HbA_1c_ Level

For HbA_1c_ level, there was evidence of a differential change over time between the 2 study groups, as indicated by a significant study group and time interaction (*P* = .009). At 6 months, the first intervention group had a slight mean increase in HbA_1c_ level of 0.1% (95% CI, −0.05% to 0.4%; *P* = .18), while the delayed intervention group had a similarly slight mean increase of 0.4% (95% CI, 0.2%-0.6%; *P* < .001). The difference between groups at 6 months was statistically significant (0.3%; 95% CI, 0.03%-0.6%; *P* = .03) (Table 2, Figure 2C, and Figure 3C).

### Secondary Outcomes

The mixed-effects models for body weight and BMI showed significant interactions between study group and time (*P* = .001 and *P* = .003, respectively). For BMI, at 6 months, the first intervention group had a mean reduction of 1.1 (95% CI, 0.4-1.7; *P* = .003), while the delayed intervention group had a mean increase of 0.3 (95% CI, −1.5 to 2.0; *P* = .75) (Table 2, Figure 2D, and Figure 3D). For body weight, at 6 months, the first intervention group had a mean reduction of 4.9 (95% CI, 1.0-8.7) kg (*P* = .01), while the delayed intervention group had a mean reduction of 3.3 (95% CI, 0.01-6.5) kg (*P* = .051) (Table 2, Figure 2E, and Figure 3E). For the supplemental analyses, parallel line plots for individual participants’ systolic BP, diastolic BP, HbA_1c_ levels, body weight, and BMI over time are shown in eFigures 1 to 5 in Supplement 2.

## Discussion

In this pilot cluster-randomized clinical trial among African immigrant adults with cardiometabolic risk factors, we found that a 6-month culturally tailored, virtual lifestyle intervention resulted in clinically meaningful improvements in systolic and diastolic BP, body weight, and BMI compared with the delayed intervention. The Afro-DPP program is one of the few pragmatic studies to adapt and test the effect of the National DPP program to improve the CMH of African immigrants. The observed reductions in BP and weight suggest that adapting evidence-based lifestyle programs, by incorporating cultural elements and leveraging virtual platforms, may be an effective strategy for improving CMH outcomes in this high-risk population. The findings of this pilot cluster-randomized trial provide preliminary evidence of the feasibility and effectiveness of adapting and delivering culturally sensitive, virtual multicomponent interventions that include lifestyle coaching, remote BP monitoring, and weight monitoring to improve the CMH of African immigrants.

To our knowledge, this is the first CMH intervention that involved cultural tailoring of the DPP curriculum for African immigrants. Our findings are consistent with previous studies demonstrating the benefits of lifestyle interventions adapted from the DPP in improving cardiometabolic profiles among racial and ethnic minoritized groups.^16,21^ A recent randomized clinical trial^22^ showed that a digital DPP produced significantly greater reductions in HbA_1c_ levels and body weight compared with enhanced standard care among participants with prediabetes, highlighting the potential for widespread dissemination and impact of digital health interventions in preventive health care services. A community-based translation of the DPP among underserved populations^23^ reported a mean weight reduction of 4.4% and modest improvements in HbA_1c_ levels. Similarly, a culturally tailored DPP intervention delivered virtually to low-income, Spanish-speaking Latino individuals^24^ significantly reduced weight and HbA_1c_ and cholesterol levels. However, unlike earlier community-based trials conducted predominantly among Black or Hispanic populations, our study uniquely focused on African immigrants and used a virtual delivery model augmented with remote monitoring of BP and body composition measures.

While the changes in BP, weight, and BMI were promising, the Afro-DPP intervention did not result in statistically significant reductions in HbA_1c_ levels at 6 months. This result may be due to the intervention’s relatively short duration or the fact that a substantial proportion of participants did not have prediabetes at baseline. Longer-term follow-up may be warranted to fully evaluate the impact on glycemic control. Furthermore, integrating more intensive pharmacological management alongside lifestyle modifications could potentially yield greater improvements in HbA_1c_ levels.^25,26^ Also, future studies should include systematic tracking of individual session attendance to better assess intervention dose effects.

The positive outcomes observed in this study may be attributed to several factors. First, our intervention was developed through extensive community engagement and tailored to incorporate cultural values, beliefs, and dietary preferences specific to African immigrant communities.^27^ Engaging faith-based organizations and community leaders for the initial phases enhances trust, ownership, and commitment, likely contributing to the high retention and adherence rates observed.^20^ Second, using a lifestyle coach of African descent who could provide culturally congruent counseling and relatable examples may have facilitated a better understanding and adoption of the recommended lifestyle modifications.^18^ African immigrant populations face the challenge of navigating dietary choices in foreign environments and perceive available food options as unhealthy, while their food choices before migrating may be limited and expensive in the host country.^28^ Incorporating culturally appropriate dietary recommendations focused on familiar food choices, along with a culturally congruent coaching approach, may have enabled participants to make informed decisions about healthy food options.

A unique aspect of our study was the incorporation of a virtual component, which significantly enhanced accessibility and participant engagement. While virtual sessions were initially prompted by COVID-19 pandemic restrictions, they effectively mitigated barriers related to transportation and scheduling for the coaching sessions. The virtual platform was complemented by one-on-one counseling sessions during the data collection, facilitating ongoing follow-up and clarification of concepts. At the core of the Afro-DPP program was the deployment of digital platforms and apps enabling remote monitoring of BP and weight, providing convenience and personalized feedback throughout the intervention. Despite initial connectivity challenges, periodic troubleshooting enabled participants to consistently monitor their health measures, likely contributing to the observed improvements in BP and weight. The virtual group sessions, complemented by individual remote monitoring, enabled continuous engagement, personalized feedback, and real-time adjustments, overcoming common barriers related to digital literacy, access, and transportation. Although earlier studies have adopted the National DPP program, particularly in faith-based settings, only a few have incorporated digital platforms despite their known convenience, accessibility, and positive impact on health outcomes.^22,24,29^

Our study provides valuable preliminary evidence supporting the feasibility and potential efficacy of a culturally tailored, virtual lifestyle intervention in improving key CMH indicators among African immigrants. These findings pave the way for larger, adequately powered definitive trials with extended follow-up to comprehensively evaluate such interventions’ long-term clinical impacts, cost-effectiveness, and scalability in mitigating cardiometabolic disparities in this rapidly growing minoritized population. Exploring strategies to enhance engagement and retention, such as incorporating mobile health technologies, peer support networks, and incentive-based approaches, is important.^30^

### Strengths and Limitations

This study has a number of strengths. The retention rate observed in our study was higher than that reported in earlier community-based studies,^24,29^ suggesting that our intervention maintained participant engagement despite the limited in-person interaction. In addition, there was a high proportion of female participants in the study, highlighting the success of our efforts to improve participation and enrollment of women into trials.

Our study also has several limitations that should be considered when interpreting the results. First, the relatively short follow-up period of 6 months limits our ability to determine whether the observed reductions in weight, HbA_1c_ levels, and BP would be sustained long term. Future studies with longer follow-up periods are needed to assess the long-term effectiveness of the intervention. Second, since the study was a pilot, the small sample size may have limited our power to detect significant differences in some outcomes. However, the sample size was appropriate for a pilot study designed to test a culturally tailored intervention’s feasibility and preliminary effectiveness among African immigrants. We acknowledge that the COVID-19 pandemic restrictions posed additional challenges for in-person meetings and home visit follow-ups, as reported in other DPP studies. In-person visits provide opportunities to enhance intervention retention, allow peer-to-peer interaction, and facilitate discussion-oriented activities that may have a greater impact on behavior change. We acknowledge that remote monitoring in the delayed intervention group may have influenced health behaviors even before their formal intervention began. This form of attention control allows us to better isolate the effects of the lifestyle intervention components while recognizing that monitoring itself may have therapeutic effects. Last, we note that the imbalance in key demographic characteristics, such as the length of stay in the US, may also impact the generalizability of our findings.

## Conclusions

The preliminary findings from the Afro-DPP for African immigrants at risk for cardiovascular disease demonstrate that a culturally tailored lifestyle intervention may effectively improve the CMH of this population. Integrating digital health solutions, remote monitoring, and culturally sensitive lifestyle coaching showed promise in promoting self-management and adherence to the intervention. These findings suggest potential avenues for further research and broader implementation of culturally sensitive interventions to mitigate cardiovascular risk factors among African immigrants.

## Notes

Supplement 1.
Trial Protocol

Supplement 2. **eFigure 1.** Model-Based Means for Systolic Diastolic Blood Pressure by Study Group Over Time **eFigure 2.** Model-Based Means for Diastolic Blood Pressure by Study Group Over Time **eFigure 3.** Model-Based Means for Hemoglobin A_1c_ by Study Group Over Time **eFigure 4.** Model-Based Means for Body Weight by Study Group Over Time **eFigure 5.** Model-Based Means for BMI by Study Group Over Time

Supplement 3.
Data Sharing Statement
