# Supplementary material for: A Virtual Cardiometabolic Health Program Among African Immigrants in the US: A Pilot Cluster-Randomized Clinical Trial
Source: JAMA Netw Open. 2025 Mar 4;8(3):e2462559. doi: 10.1001/jamanetworkopen.2024.62559 (PMC11880947; doi:10.1001/jamanetworkopen.2024.62559)
Supplement: Supplement 1. — Trial Protocol [file jamanetwopen-e2462559-s001.pdf]

SUPPLEMENT 1

STUDY PROTOCOL

The Afro-DPP Program: A virtual cardiometabolic health program to improve blood pressure and glycemic control among African Immigrants in the US: A Pilot Randomized Clinical Trial

Funding: National Institute of Nursing Research (NINR)

Principal Investigators
Yvonne Commodore-Mensah PhD, MPH, RN
Oluwabunmi Ogungbe, PhD, MPH, RN

Co-Investigators

This supplement contains the following items:

1. Original protocol and original statistical analysis plan.
2. Final protocol, final statistical analysis plan, and summary of changes

Original Version Approved November 30, 2020

Table of Content

Contents

Abstract 4
Objectives 4
Background 5
  Preliminary Data 5
Study Procedures 6
Data Management 9
Inclusion/Exclusion Criteria 10
Study Statistics 11
Risks 12
Benefits 13

Abstract

Black people and immigrants in the United States (US) are disproportionately affected by poor cardiometabolic health (CMH), defined by risk factors for cardiovascular disease (CVD) and type 2 diabetes (T2D).

African immigrants are among the fastest-growing segments of black immigrants in the U.S., increasing 137% from 2000 to 2013. More than a third are from West specifically Ghana and Nigeria. There are significant gaps in knowledge on African immigrants in the U.S. because "Blacks" are often studied as a homogeneous racial group in health disparities research.[2] In the “Afro-Cardiac Study”[5], a cross-sectional study (N=253) conducted by our team, 88% of the African immigrants were overweight/obese, 40% had hypertension, 16% had T2D, and only 52% had health insurance. In our ongoing study, “The African Immigrant Health Study”, only 60% of the 395 participants had health insurance. Participants from both studies were recruited from faith-based settings in the Baltimore-Washington, D.C. area.

To date, there are no existing community-based programs to improve the CMH of African immigrants at risk of developing CVD and T2D. Due to our prior success engaging African immigrants in faith-based settings[7], we propose a “Virtual Cardiometabolic Health Program” among African immigrants to build on our previous cross-sectional and community-engaged research with consideration of cultural values, practices, beliefs, and faith.

Digital health interventions, defined as the different ways in which digital and mobile technologies are being used to support health needs, are promising complements to lifestyle interventions.[8] Digital health interventions (i.e. interventions delivered via digital technologies such as smartphones, websites, and text messaging) are effective, cost-effective, safe, and scalable which promote healthy behavior to promote CMH.[9]

The proposed study will address the CMH of community-dwelling African immigrants who have multiple cardiometabolic risk factors including hypertension, prediabetes, and overweight/obesity. We seek to improve CMH by offering a **virtual program** that will improve access to therapeutic lifestyle interventions amid the COVID-19 pandemic. We hypothesize that a combination of lifestyle intervention with a culturally specific curriculum and patient monitoring will improve participants’ blood pressure management and lead to a reduction in body weight.

Objectives

We seek to address the following specific aims:

Aim 1: To examine whether a virtual, culturally-tailored, lifestyle intervention with remote monitoring of blood pressure and body composition (The Afro-DPP program) would reduce blood pressure and body weight compared to remote monitoring of blood pressure and body composition alone among African immigrants.

Aim 2: To examine whether The Afro-DPP program would reduce cardiovascular disease risk, body fat percentage, and body mass index compared to remote monitoring of blood pressure and body composition alone among African immigrants

The primary outcomes are the reduction in blood pressure and body weight. We will also assess secondary outcomes including the reduction in global CVD risk, body fat percentage, and body mass index over 12 months. Global CVD risk, which will be calculated with the BMI-based CVD risk score, and body fat percentage are considered proximal outcomes. The distal outcomes that are unlikely to be observed in the timeframe of the pilot study include improvement in positive affect/well-being and quality of life (QoL). We

will use the PROMIS global health measure to rigorously assess the QoL of participants to determine the impact of the intervention on patient-reported outcomes.

Background

African immigrants are one of the fastest-growing segments of black immigrants in the U.S., increasing 137% from 2000 to 2013. More than a third are from West specifically Ghana and Nigeria. There are significant gaps in knowledge on African immigrants in the U.S. because "Blacks" are often studied as a homogeneous racial group in health disparities research.[2]

The limited data that exist on African immigrants suggest that they are distinct from their African American counterparts but could provide valuable insight into health disparities among Black people. The triple epidemics of obesity, T2D, and hypertension have taken hold in Africa with Africans acquiring CVD risk at higher rates before migration.[3] Hence, current African immigrants may have poorer CMH than previous generations. A prior clinical study at the National Institute of Health found that African immigrants were more likely to have prediabetes or diabetes compared to African Americans, and they were also more unaware of their risk.[4]

Despite well-publicized guidelines on the appropriate management of CVD and T2D, the implementation of risk-reducing practices remains poor. The National Diabetes Prevention Program (DPP) has shown that lifestyle change involving a lifestyle coach, CDC-approved curriculum, or group support is effective for improving CMH and reducing T2D risk by 58% in a cost-effective manner.[6,11] The National DPP Lifestyle Intervention curriculum is based on the curriculum from the DPP trial.[6] However, evidence-based programs such as the DPP have not been adapted to African immigrants, a vulnerable population that is predisposed to poor CMH. The evidence base supporting self-monitored blood pressure monitoring (SMBP), also known as home blood pressure monitoring, for patients with hypertension is strong.[13] SMBP plus additional support, defined as one-on-one counseling, web-based or telephonic support, and educational classes, leads to significant improvement in systolic blood pressure (SBP) (mean net reduction of 1.6-8.5 mm Hg) and diastolic blood pressure (DBP) (the mean net reduction of 1.9-4.4 mm Hg).[13]

Preliminary Data

In the “Afro-Cardiac Study”[5], a cross-sectional study on African immigrants in the United States (N=253) conducted by our team, 88% were overweight/obese, 40% had hypertension, 16% had T2D, and only 52% had health insurance. In our ongoing study, “The African Immigrant Health Study”, only 60% of the 395 participants had health insurance. Participants from both studies were recruited from faith-based settings in the Baltimore-Washington, D.C. area. To date, there are no existing community-based programs to improve the CMH of African immigrants

The proposed intervention is informed by the PEN 3 Model which was originally developed as a framework for designing culturally-appropriate health promotion and disease prevention interventions in African countries.[14] The PEN 3 Model has also been successfully applied to other health promotion interventions targeting African Americans including the DPP. [15] The PEN-3 model completes cultural adaptions in two phases that support community input on the appropriate adaptation elements. The first phase, assessment, involves information gathering to learn about the community and its perspective, the resources that promote or inhibit behavioral change, and the roles that the social network plays in behavioral change. We have completed Phase 1 of this process in our recent qualitative work among African immigrants. We have recently conducted seven focus groups with African immigrants from Ghana, Nigeria, Liberia, and Sierra Leone and will use the data collected in the focus groups to inform the co-creation of this program in faith-based settings. Community-based management of CVD risk factors and T2D has been previously implemented in ethnic/minority groups such as African Americans[16,17], Hispanic immigrants[18,19,] and faith-based settings.[20,21] However, it is unclear if these strategies can be translated to African immigrants in faith-based settings. We

are proposing an intervention that would potentially improve access to a cost-effective intervention in a familiar environment with strong social networks.

Digital health interventions, defined as the different ways in which digital and mobile technologies are being used to support health needs, are promising complements to lifestyle interventions.[8] Digital health interventions (i.e. interventions delivered via digital technologies such as smartphones, websites, and text messaging) are effective, cost-effective, safe, and scalable which promote healthy behavior to promote CMH. [9]

Study Procedures

a. Study design, including the sequence and timing of study procedures

We will use a non-equivalent control group design to test the effectiveness of the intervention at two African churches in the Baltimore, Washington, D.C. area. The two churches will be randomly assigned to the intervention or delayed intervention group. At the end of a 6-month follow-up period, the control church will receive the intervention (delayed control group). We have opted for this design approach to allow both churches to benefit from the proposed intervention address the ethical concerns and maintain good retention rates in the trial.

Screening and Recruitment

We will recruit a total of 60 participants,30 participants at each of the two African churches. Since the intervention includes a digital health component, participants who do not have smartphones will receive loaner phones to ensure equity in access to the program. The research team has already attained written authorization to contact participants from the African Immigrant Health Study (IRB 00129394). Previous participants will be contacted to see if they are interested in joining the Afro-DPP Program. We will also engage leaders of organizations that have participated in our previous studies to assist with engaging their constituents and reaching our targeted sample size.

Informed Consent

Once a person at one of the churches expresses interest in the study, a consent designee of the research study team will contact her/him by email or phone to schedule a time to obtain informed consent and discuss study activities.

Study procedures

All participants will receive a Bluetooth-enabled digital scale (Omron Model: BCM-500) that measures body composition including Body Weight, Body Fat percentage, Visceral Fat, Skeletal Muscle percentage, Resting Metabolism, and Body Mass Index. A Bluetooth-enabled blood pressure monitor (Omron Model: BP7250) will also be distributed to all participants. All participants will download the Omron Connect app which will allow them to sync their blood pressure readings and body composition readings into the app. At the baseline visit, the participants would be educated on how to use these devices to take physical measurements at home. The research team will access these readings to monitor study outcomes and participants' progress during the follow-up period.

The intervention curriculum will be delivered by a certified DPP Lifestyle Coach who is of African descent over two 6-month periods. Participants in the *1st intervention group* will immediately begin 6 months of the adapted DPP lifestyle intervention with a Lifestyle Coaches and remote monitoring of blood pressure and body composition. This will be followed by a 6-month observation period where the intervention will be withdrawn. In this period, participants will be evaluated for the maintenance of lifestyle changes. Participants in the *delayed intervention group* will receive remote monitoring of blood pressure and body composition for the 1st 6 months and then will receive the adapted DPP lifestyle intervention after 6 months.The multicomponent intervention will focus on intensive lifestyle modification delivered by the Lifestyle Coach. The Lifestyle Coach of African origin will be responsible for delivering the intensive lifestyle intervention and implementing the adapted DPP curriculum. The curriculum from the DPP program will be culturally adapted with input from healthcare professionals and community leaders who

are also African immigrants. Our goal is to adapt the DPP to African immigrants with consideration of African cultural values, practices, beliefs, and faith by including culturally appropriate examples in the educational content to make the information more acceptable to the participants. For instance, the module titled “Shop and Cook to Prevent T2D” will be adapted to “Shop and Cook to Promote Cardiometabolic Health” and include examples of African foods that are heart-healthy. The CDC-developed curriculum includes a facilitator’s guide and participant handouts which will be used by the Lifestyle Coach to implement the lifestyle intervention to all participants. The adapted DPP curriculum is described briefly in **Table 1**.

TABLE 1. Adapted DPP Curriculum

Original Sessions	Modified Sessions	Week	Adaptation
1. Welcome to the National DPP Program	Welcome to the National DPP Program	1	-
2. Be a Fat and Calorie Detective	Fats: Detect and Eliminate	3	Incorporate examples of sources of fats in African foods such as palm oil
3. Three Ways to Eat Less Fat and Fewer Calories			
4. Healthy Eating	Healthy Eating the African Way: Part 1	5	Provide examples of healthier versions of African food
**2.** Move those Muscles	Be Active: Move your body	7	Incorporate African dance lessons in the session
**3.** Being Active – A Way of Life			
**4.** Tip the Calorie Balance	Finding balance	9	-
**5.** Take Charge of What’s Around You			
**6.** Problem Solving	Same as Original	11	-
**7.** Four Keys for Healthy Eating Out			
**8.** Talk Back to Negative Thoughts	Taking Charge	13	Combine sessions on facing mental challenges related to healthy lifestyle practices
**9.** The Slippery Slope of Lifestyle Change			
**10.** Jump Start Your Activity Plan	Get to it	15	
**11.** Make Social Cue Work for You			
**12.** You Can Manage Stress	Managing Stress by Faith	17	Discuss unique stressors that African immigrants face<br><br>Discuss mindfulness, meditation, and exercise for stress reduction
**13.** Ways to Stay Motivated			
**14.** Fats- Saturated, Unsaturated, and Trans Fat	Cook for life	19	-
**15.** Food Preparation and Recipe Modification			
**16.** Healthy Eating – Taking it One Meal at a Time	Healthy Eating the African Way: Part 2	21	Emphasize healthier African foods
**17.** Healthy Eating with Variety and Balance			
**18.** More Volume, Fewer Calories			
**19.** Staying on Top of Physical Activity	Keeping fit: the African way	23	Emphasize different options for meeting exercise recommendations
**20.** Stepping up to Physical Activity			
**21.** Balance Your Thoughts for Long-Term Maintenance	Face the Giants like King David	25	-

**22.** Handling Holidays, Vacations, and Special Events			
**23.** Preventing Relapse			
**24.** Stress and Time Management	Stress and your Cardiometabolic Health	27	-
**25.** Heart Health			
**26.** A Closer Look at Type 2 Diabetes			
**27.** Final Session: Looking Back and Looking Forward	Final Session: Looking Back and Looking Forward	30	-

At each in-person follow-up visit, blood pressure will be measured 3 times at intervals of one minute or longer using the Omron Model: BP7250, and the average of the three recorded. Hemoglobin A1c will be measured using the point-of-care testing system described in section 6 below. BMI (height and weight) will be measured using Omron Model: BCM-500. The follow-up questionnaires will be completed online by participants to reduce the amount of time spent at the church location.

Data Collection

Data collection for this study will be performed by two trained Research Assistants. A study protocol will be developed and utilized to ensure standardization of measurements. **Table 2** describes the measures which will be obtained for this study. We selected measures based on their psychometric properties as well their alignment with common data elements which have been identified by the Common Data Repository for Nursing Science and are being used in other pilot studies in the PROMOTE Center**.**

Table 2. Participant measures by Study Visit

	Baseline	1 month	3 months	6 months	12 months
Survey Measures					
**Demographic Information (BRICS NINR Demographics)**	X				
**Health Behaviors (smoking, alcohol use, diet, physical activity)**	X				X
BRICS NINR Social Determinants of Health	X				X
Health History	X				
**PROMIS Global Health measure v1**[26]	X			X	X
**PROMIS-Fatigue Short form 6a**[27]	X			X	X
Brief Resilience Scale[28]	X			X	X
PROMIS Depression Short Form 6a[29]	X			X	X
Perceived Stress Scale [30]	X			X	X
ENRICHD Social Support Inventory [31]	X				X
The CoRonavIruS Health Impact Survey (CRISIS)[32]	X		X	X	X
Rand Health Care Short Form Health Survey (SF-36)	X			X	X
Physical and Biochemical Measures					
Systolic Blood Pressure	X	X	X	X	X
Diastolic Blood Pressure	X	X	X	X	X
Body Weight	X	X	X	X	X
Body Mass Index	X	X	X	X	X
Body Fat Percentage	X	X	X	X	X
Hemoglobin A1c	X		X	X	X
**Bold: Common Data Elements in the Common Data Repository for Nursing Science**					

Data Management

We will use REDCap for the study database. Data from screening and intervention visits will be entered on standardized forms or directly into the REDCap database. The research assistants will check the standardized forms for completeness and appropriateness. During data entry, the participants will be assigned a unique identifying number that will be separated from their names and other identifying information and kept in a separate, secure data file. The confidentiality of participants will be ensured by using the unique identifying numbers attached to the participant's responses. Only the study team members will have access to this file of identifying information for research purposes only. Identifying information, including names, will be kept separate from the participant's survey responses and protected in a secure, password-protected, and HIPAA-compliant server or a secure, locked filing cabinet in the PI's locked office. Reporting of results will not involve the identification of participants as data will be presented in collective or aggregate form.

The duration of the study is 12 months and will have a total of 5 in-person follow-up time points. The project timeline is outlined in **Table 3**.

TABLE 3: Project Timeline

	**Year 1**				**Year 2**			
	**August 2020-July 2021**				**August 2021-July 2022**			
	**Aug-Oct**	**Nov-Jan**	**Feb-Apr**	**May-Jul**	**Aug-Oct**	**Nov-Jan**	**Feb-Apr**	**May-Jul**
**Phase I: Development Phase**								
Focus group with African immigrants		■						
IRB application		■						
Cultural adaptation of educational materials and DPP curriculum		■						
Finalize study materials including intervention and data collection protocols	■	■						
Recruitment and training of Lifestyle Coach and Research Assistants	■	■						
**Phase II: Implementation Phase**								
Baseline data collection		■						
Intervention delivery Site 1(N=30)			■	■				
Maintenance Phase Site 1					■	■		
Intervention delivery Site 2 (N=30)					■	■		
Monthly follow-up visits			■	■	■	■		
Quality Assessment Monitoring			■	■	■	■		
Post-intervention data collection						■		
**Phase III: Evaluation and Planning for R01 Submission**								
Data analysis							■	
Preparation of manuscripts and abstracts reporting on study methods and findings								■
Prepare and submit proposals for funding for the next phase of research								■

Study duration and number of study visits required of research participants.

The duration of this study is 12 months. There will be 5 in-person study visits at both churches. The study visits will take place at the Baseline, 1-month, 3-month, 6-month, and 12-month points of the study in private rooms at the churches. The study team and participants will observe COVID-19 Protocols during the in-person visits.

Blinding, including justification for blinding or not blinding the trial, if applicable.

We will use a non-equivalent control group to test the effectiveness of the intervention. The intervention group will receive the intervention at the start of the study whereas the delayed control group will receive the intervention after 6 months. While the participants will be blinded to which group they are in, study staff, data collectors, and analysts will not be blinded given the interactive nature of the study.

Justification for inclusion of a placebo or non-treatment group.

We will use a non-equivalent control group design to test the effectiveness of the intervention at two African churches in the Baltimore, Washington, D.C. area. The two churches will be randomly assigned to the intervention or control group. At the end of a 6-month follow-up period, the control church will receive the intervention (delayed control group). We have opted for this design approach to allow both churches to benefit from the proposed intervention address the ethical concerns and maintain good retention rates in the trial.

Definition of treatment failure or participant removal criteria.

Participants will be excluded from the study if a cognitive challenge or serious illness arises that would interfere with study participation. If a participant does not attend at least 18 out of the 36 total DPP sessions, then they would have failed to complete study participation.

Description of what happens to participants receiving therapy when the study ends or if a participant's participation in the study ends prematurely.

When the study ends, participants will keep study materials such as the scale and the blood pressure device, however, loaner smartphones will be returned. If a participant chooses to withdraw from the research study, study personnel will inform the participant that compensation from the study will be withheld, due to prematurely ending participation.

Inclusion/Exclusion Criteria

Inclusion criteria:

We will include:

- African immigrants who are aged 25-75 years
- Participants who report being uninsured or have no access to a healthcare provider
- Have at least two of the following chronic conditions:
    - Body-mass index ≥ 25 kg/m^2
    - Self-reported fasting plasma glucose of 95 to 125 mg/d or HbA1c of 5.7-6.5% in the past 6 months
    - Systolic blood pressure ≥140 mmHg or diastolic blood pressure ≥ 90 mmHg

Exclusion criteria:

We will exclude:

- Participants who cannot communicate in English
- Participants who have cognitive challenges that would restrict them from participation
- Participants who have any serious illness that would interfere with participation
- Participants who are not members of the churches that are involved in this study

Devices

The rationale for choosing the drug and dose or for choosing the device to be used.

Hemoglobin A1CNOW®+ Point of Care System: A hemoglobin A1c (HbA1c) test that measures the amount of blood sugar (glucose concentrations) attached to hemoglobin will be obtained with a fingerstick (capillary blood) and measured using a point of care testing instrument (POCT)- A1CNOW®+. The accuracy and precision of the fingerstick measurements by using A1CNOW®+ have been examined in previous studies. [31] Although laboratory measurements have traditionally been used for the evaluation of biochemical markers of Diabetes risk, this is only convenient in a hospital-based setting. With this community-based study, a laboratory-based approach has the limitations of transporting and storing blood samples, delay in obtaining the results, and the need to attempt to contact participants again after the results become available. Hence, this POCT meets the relevant National Glycohemoglobin Standardization Program (NGSP) certification, Diabetes Control and Complications Trial (DCCT), and the United Kingdom Prospective Diabetes Study (UKPDS) which established the direct relationships between HbA1c levels and outcome risks in patients with diabetes. The A1C results ranged from 5.0 % A1C to 12.8 % A1C, with a mean of 7.3 % A1C. Accuracy of A1CNow+, with fingerstick samples, was, on average, 99%. This means that, on average, a true 7.0% A1C could read approximately 6.9% A1C. An individual A1CNow+ result may differ by as much as -1.0% A1C to +0.8% A1C from the true result. This represents the 95% confidence limits of a Bland-Altman plot. [32] This will allow for the provision of immediate counseling that can lead to behavior changes and healthier lifestyles. The Hemoglobin A1c results would be shared with the participants on a report card.

Study Statistics

a. **Primary outcome variable.**

The primary outcome variable is a reduction in blood pressure and body weight.

b. **Secondary outcome variables.**

The secondary outcome variable is the reduction in global CVD risk, body fat percentage, and body mass index.

c. **Statistical plan including sample size justification and interim data analysis.**

We plan to recruit 30 participants from each of the two churches. Given that this is a pilot study, we will only have sufficient power to detect large effect sizes. Based on the results of the Afro-Cardiac Study[5], we assume the rate of elevated CVD risk assessed with the Pooled Cohort Equation will be 28%. With statistical power of 0.80, alpha of 0.05, and 30 participants per arm, we will be able to detect significant differences if groups differ by 25% at a 6-month follow-up. The effect sizes observed in this pilot study will be used to conduct power analyses to inform sample size estimates for a future randomized controlled effectiveness trial. The planned enrollment is described in **Table 4.**

TABLE 4: Planned Enrollment Report					
**Racial Categories**	**Ethnic Categories**				
	**Not Hispanic or Latino**		**Hispanic or Latino**		**Total**
	**Female**	**Male**	**Female**	**Male**	
American Indian/ Alaska Native	-	-	-	-	-
Asian	-	-	-	-	-
Native Hawaiian or Other Pacific Islander	-	-	-	-	-
Black or African American	30	30	-	-	-
White	-	-	-	-	-
More than One Race	-	-	-	-	-
**Total**	**30**	**30**			**60**

Early stopping rules.
Though minimal risks are involved within this research study; risk events, problems, and protocol deviations will be reported by the Principal Investigator directly to the IRB.

Risks

Medical risks, listing all procedures, their major and minor risks, and expected frequency.

Minor risks involving physical activities and dietary changes may include an increase in the risk of injury or affect the cardiovascular system. Participants will be encouraged to recognize their capabilities and limits before being involved in physical activities scheduled throughout the program. The potential risks are associated with local bleeding from the site of the finger stick. However, since capillary blood samples instead of venous samples will be obtained, the risk of gross bleeding will be lowered.

Steps were taken to minimize the risks.
Recruitment and Informed consent

Informed consent will be obtained from all study participants. The informed consent document and the explanation of the study will make it clear that participation in the proposed study is completely voluntary and can be stopped at any point and for any reason.
In addition to ensuring that participants are well-informed on the risks associated with the study, only trained research assistants will conduct the capillary blood draws and strict adherence to universal precautions will be maintained. It will be emphasized the tests are not diagnostic and follow-up is necessary for an actual diagnosis of hypertension and diabetes. Participants will be referred to their primary care providers or if uninsured, referred to community health services if they have blood pressures greater than 180/120 mmHg or Hemoglobin A1c levels greater than 6.5%. 911 would be called in the case of any emergency.

Plan for reporting unanticipated problems or study deviations.

We do not expect any deviations from the study. However, any deviations from the study plan or unanticipated problems will be reported immediately to the JHM IRB.

Legal risks such as the risks that would be associated with breach of confidentiality.

During data entry, the participants will be assigned a unique identifying number that will be separated from their names and other identifying information and kept in a separate, secure data file. The confidentiality of participants will be ensured by the use of the unique identifying numbers in the participants' responses. Only the study team members will have access to this file of identifying information for research purposes only. Identifying information, including names, will be kept separate from the participant's survey

responses and protected in a secure, password-protected, and HIPAA-compliant server or a secure, locked filing cabinet in the PI's locked office. Reporting of results will not involve the identification of participants as data will be presented in collective or aggregate form.

Financial risks to the participants.

There will be no financial risks to the participants. Study materials such as the Bluetooth-enabled digital scale (Omron Model: BCM-500) and a Bluetooth-enabled blood pressure monitor (Omron Model: BP7250) will be distributed to all participants. The Omron Connect application is free for all users.

Benefits
Description of the probable benefits for the participant and society.

We hope that the group-based lifestyle intervention will improve the presence of positive emotions in daily activities, participation in society, satisfying relationships, and overall life satisfaction.

Payment and Remuneration
Detail compensation for participants including possible total compensation, proposed bonus, and any proposed reductions or penalties for not completing the protocol.

During the Baseline and first-month research visits, all participants enrolled in this research study will receive a compensation of $30.00 (thirty dollars) upon completing the Baseline and first-month research visits.

During the third-month research visit, all participants enrolled in this research study will receive a compensation of $40.00 (forty dollars) upon completing the third-month research visit.

During the sixth month and twelfth (last) month of research visits, all participants enrolled in this research study will receive a compensation of $75.00 (seventy-five dollars) upon completing the sixth month and twelfth-month research visits. The total potential payment for all participation in the study equals $250.00 (the sum of all five research visits)

Costs

Detail costs of study procedure(s) drug (s) or substance(s) to participants and identify who will pay for them.
Study participants recruited and enrolled will not be responsible for any study-related costs.

BIBLIOGRAPHY AND REFERENCES CITED

1. Dickman SL, Himmelstein DU, Woolhandler S. Inequality and the health-care system in the USA. *Lancet*. 2017;389(10077):1431-1441. doi: S0140-6736(17)30398-7 [pii].

2. Commodore-Mensah Y, Himmelfarb CD, Agyemang C, Sumner AE. Cardiometabolic health in African immigrants to the United States: A call to re-examine research on African-descent populations. *Ethn Dis*. 2015;25(3):373-380. doi: 10.18865/ed.25.3.373 [doi].

3. Commodore-Mensah Y, Samuel LJ, Dennison-Himmelfarb CR, Agyemang C. Hypertension and overweight/obesity in Ghanaians and Nigerians living in West Africa and industrialized countries: A systematic review. *J Hypertens*. 2014;32(3):464-472. doi: 10.1097/HJH.0000000000000061 [doi].

4. O'Connor MY, Thoreson CK, Ricks M, et al. Worse cardiometabolic health in African immigrant men than African American men: Reconsideration of the healthy immigrant effect. *Metab Syndr Relat Disord*. 2014. doi: 10.1089/met.2014.0026 [doi].

5. Commodore-Mensah Y, Hill M, Allen J, et al. Sex differences in cardiovascular disease risk of Ghanaian- and Nigerian-born West African immigrants in the united states: The afro-cardiac study. *J Am Heart Assoc*. 2016;5(2):10.1161/JAHA.115.002385. doi: 10.1161/JAHA.115.002385 [doi].

6. Ratner R, Goldberg R, Haffner S, et al. Impact of intensive lifestyle and metformin therapy on cardiovascular disease risk factors in the diabetes prevention program. *Diabetes Care*. 2005;28(4):888-894. doi: 28/4/888 [pii].

7. Commodore-Mensah Y, Turkson-Ocran RA, Nmezi NA, et al. Commentary: Engaging African immigrants in research experiences and lessons from the field. *Ethn Dis*. 2019;29(4):617-622. doi: 10.18865/ed.29.4.617 [doi]

8. World Health Organization(WHO). Classification of digital health interventions v1.0: A shared language to describe the uses of digital technology for health. . 2018.

9. Widmer RJ, Collins NM, Collins CS, West CP, Lerman LO, Lerman A. Digital health interventions for the prevention of cardiovascular disease: A systematic review and meta-analysis. *Mayo Clin Proc*. 2015;90(4):469-480. doi: 10.1016/j.mayocp.2014.12.026 [doi].

10. Anderson M. A rising share of the U.S. black population is foreign born. Pew Research Center Web site. http://www.pewsocialtrends.org/2015/04/09/a-rising-share-of-the-u-s-black-population-is-foreign-born/. Published 2015. Updated 2015. Accessed April,3, 2016.

11. Knowler WC, Barrett-Connor E, Fowler SE, et al. Reduction in the incidence of type 2 diabetes with lifestyle intervention or metformin. *N Engl J Med*. 2002;346(6):393-403. doi: 10.1056/NEJMoa012512 [doi].

12. Allen JK, Dennison-Himmelfarb CR, Szanton SL, et al. Community outreach and cardiovascular health (COACH) trial: A randomized, controlled trial of nurse practitioner/community health worker cardiovascular disease risk reduction in urban community health centers. *Circ Cardiovasc Qual Outcomes*. 2011;4(6):595-602. doi: 10.1161/CIRCOUTCOMES.111.961573.

13. Uhlig K, Patel K, Ip S, Kitsios GD, Balk EM. Self-measured blood pressure monitoring in the management of hypertension: A systematic review and meta-analysis. *Ann Intern Med*. 2013;159(3):185-194. doi: 10.7326/0003-4819-159-3-201308060-00008 [doi].

14. Airhihenbuwa CO. A conceptual model for culturally appropriate health education programs in developing countries. *Int Q Community Health Educ*. 1990;11(1):53-62. doi: 10.2190/LPKH-PMPJ-DBW9-FP6X [doi].

15. Cowdery JE, Parker S, Thompson A. Application of the PEN-3 model in a diabetes Prevention Intervention. *Journal of Health Disparities Research and Practice*. 2010;4(1):26-41.

16. Victor RG, Lynch K, Li N, et al. A cluster-randomized trial of blood-pressure reduction in black barbershops. *N Engl J Med*. 2018;378(14):1291-1301. doi: 10.1056/NEJMoa1717250 [doi].

17. Lancaster KJ, Schoenthaler AM, Midberry SA, et al. Rationale and design of faith-based approaches in the treatment of hypertension (FAITH), a lifestyle intervention targeting blood pressure control among black church members. *Am Heart J*. 2014;167(3):301-307. doi: 10.1016/j.ahj.2013.10.026 [doi].

18. Balcazar H, Wise S, Rosenthal EL, et al. An ecological model using promotores de salud to prevent cardiovascular disease on the US-Mexico border: The HEART project. *Prev Chronic Dis*. 2012;9:E35. doi: E35 [pii].

19. Khare MM, Cursio JF, Locklin CA, Bates NJ, Loo RK. Lifestyle intervention and cardiovascular disease risk reduction in low-income Hispanic immigrant women participating in the Illinois WISEWOMAN program. *J Community Health*. 2014;39(4):737-746. doi: 10.1007/s10900-014-9820-3 [doi].

20. Brewer LC, Balls-Berry JE, Dean P, Lackore K, Jenkins S, Hayes SN. Fostering African American improvement in total health (FAITH!): An application of the American heart association's life's simple 7 among midwestern African Americans. *J Racial Ethn Health Disparities*. 2017;4(2):269-281. doi: 10.1007/s40615-016-0226-z [doi].

21. Schoenthaler AM, Lancaster KJ, Chaplin W, Butler M, Forsyth J, Ogedegbe G. Cluster randomized clinical trial of FAITH (faith-based approaches in the treatment of hypertension) in blacks. *Circ Cardiovasc Qual Outcomes*. 2018;11(10):e004691. doi: 10.1161/CIRCOUTCOMES.118.004691 [doi].

22. Commodore-Mensah Y, Sampah M, Berko C, et al. The afro-cardiac study: Cardiovascular disease risk and acculturation in West African immigrants in the united states: Rationale and study design . *J Immigr Minor Healt*. 2015.

23. American Association of Nurse Practitioners. Nurse practitioner cost effectiveness. Updated 2013.

24. Goff DC,Jr, Lloyd-Jones DM, Bennett G, et al. 2013 ACC/AHA guideline on the assessment of cardiovascular risk: A report of the American College of Cardiology/American heart association task force on practice guidelines. *Circulation*. 2013. doi: 01.cir.0000437741.48606.98 [pii].

25. Commodore-Mensah Y, Turkson-Ocran RA, Nmezi NA, et al. Commentary: Engaging African immigrants in research experiences and lessons from the field. *Ethn Dis*. 2019;29(4):617-622. doi: 10.18865/ed.29.4.617 [doi].

26. Hays RD, Bjorner JB, Revicki DA, Spritzer KL, Cella D. Development of physical and mental health summary scores from the patient-reported outcomes measurement information system (PROMIS) global items. *Qual Life Res*. 2009;18(7):873-880. doi: 10.1007/s11136-009-9496-9 [doi].

27. Cook KF, Jensen SE, Schalet BD, et al. PROMIS measures of pain, fatigue, negative affect, physical function, and social function demonstrated clinical validity across a range of chronic conditions. *J Clin Epidemiol*. 2016;73:89-102. doi: 10.1016/j.jclinepi.2015.08.038 [doi].

28. Smith BW, Dalen J, Wiggins K, Tooley E, Christopher P, Bernard J. The brief resilience scale: Assessing the ability to bounce back. *Int J Behav Med*. 2008;15(3):194-200. doi: 10.1080/10705500802222972 [doi].

29. Pilkonis PA, Choi SW, Reise SP, et al. Item banks for measuring emotional distress from the patient-reported outcomes measurement information system (PROMIS(R)): Depression, anxiety, and anger. *Assessment*. 2011;18(3):263-283. doi: 10.1177/1073191111411667 [doi].

30. Cohen S, Kamarck T, Mermelstein R. A global measure of perceived stress. *J Health Soc Behav*. 1983;24(4):385-396.

31. Mitchell PH, Powell L, Blumenthal J, et al. A short social support measure for patients recovering from myocardial infarction: The ENRICHD social support inventory. *J Cardiopulm Rehabil*. 2003;23(6):398-403.

32. The coronarivus health impact survey(CRISIS) V0.3: Adult self-report baseline current form. . 2020.
