# Supplementary material for: A Virtual Cardiometabolic Health Program Among African Immigrants in the US: A Pilot Cluster-Randomized Clinical Trial
Source: JAMA Netw Open. 2025 Mar 4;8(3):e2462559. doi: 10.1001/jamanetworkopen.2024.62559 (PMC11880947; doi:10.1001/jamanetworkopen.2024.62559)
Supplement: Supplement 2. — eFigure 1. Model-Based Means for Systolic Diastolic Blood Pressure by Study Group Over Time eFigure 2. Model-Based Means for Diastolic Blood Pressure by Study Group Over Time eFigure 3. Model-Based Means for Hemoglobin A1c by Study Group Over Time eFigure 4. Model-Based Means for Body Weight by Study Group Over Time eFigure 5. Model-Based Means for BMI by Study Group Over Time [file jamanetwopen-e2462559-s002.pdf]

Supplementary Online Content

Ogungbe O, Hinneh T, Turkson-Ocran R-AN, et al. A virtual cardiometabolic health program among African immigrants in the US: a pilot cluster-randomized clinical trial. *JAMA Netw Open.* 2025;8(3):e2452559. doi:10.1001/jamanetworkopen.2024.62559

eFigure 1. Model-Based Means for Systolic Diastolic Blood Pressure by Study Group Over Time
eFigure 2. Model-Based Means for Diastolic Blood Pressure by Study Group Over Time
eFigure 3. Model-Based Means for Hemoglobin A_{1c} by Study Group Over Time
eFigure 4. Model-Based Means for Body Weight by Study Group Over Time
eFigure 5. Model-Based Means for BMI by Study Group Over Time

This supplementary material has been provided by the authors to give readers additional information about their work.

© 2025 Ogungbe O et al. *JAMA Network Open*.

eFigure 1. Model-Based Means for Systolic Diastolic Blood Pressure by Study Group Over Time

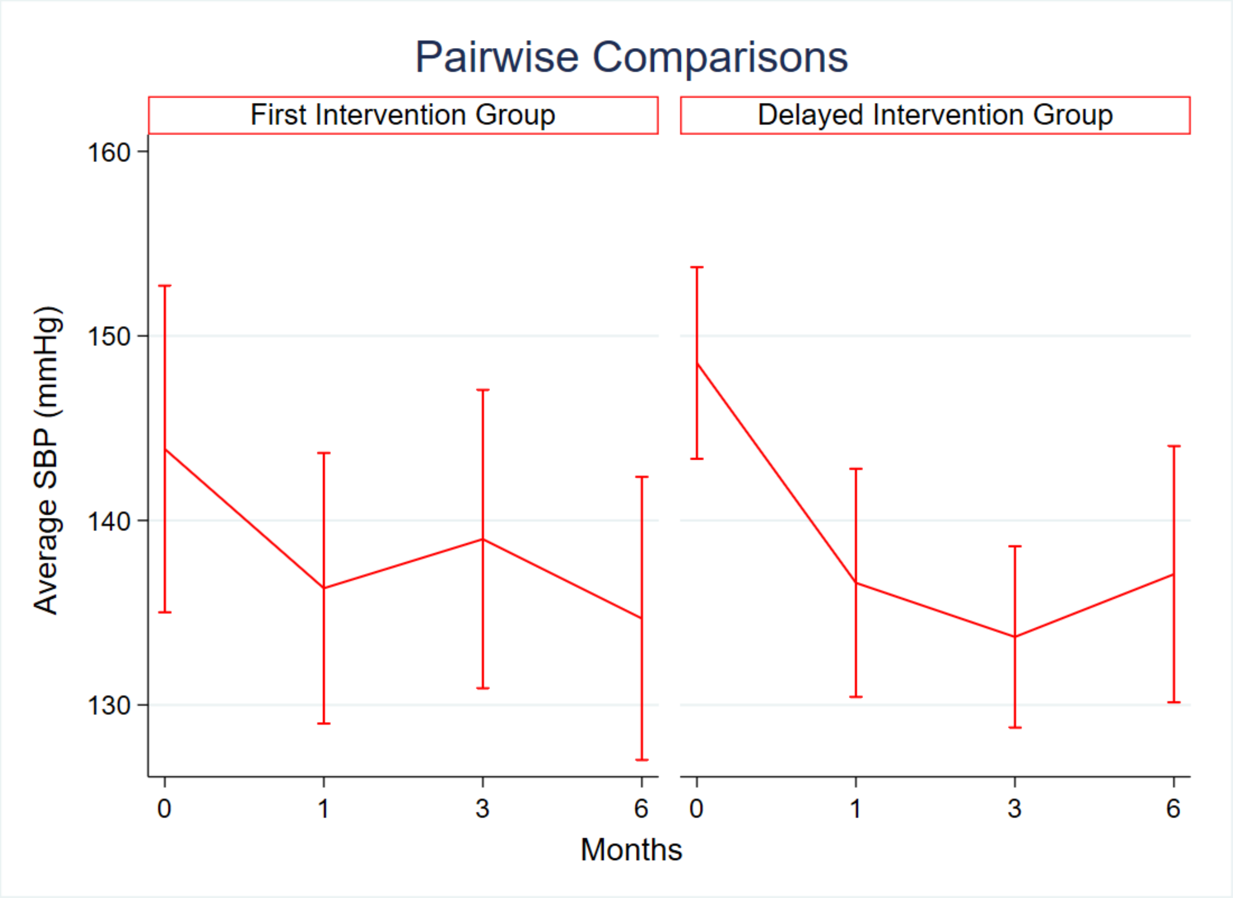

© 2025 Ogungbe O et al. *JAMA Network Open*.

eFigure 2. Model-Based Means for Diastolic Blood Pressure by Study Group Over Time

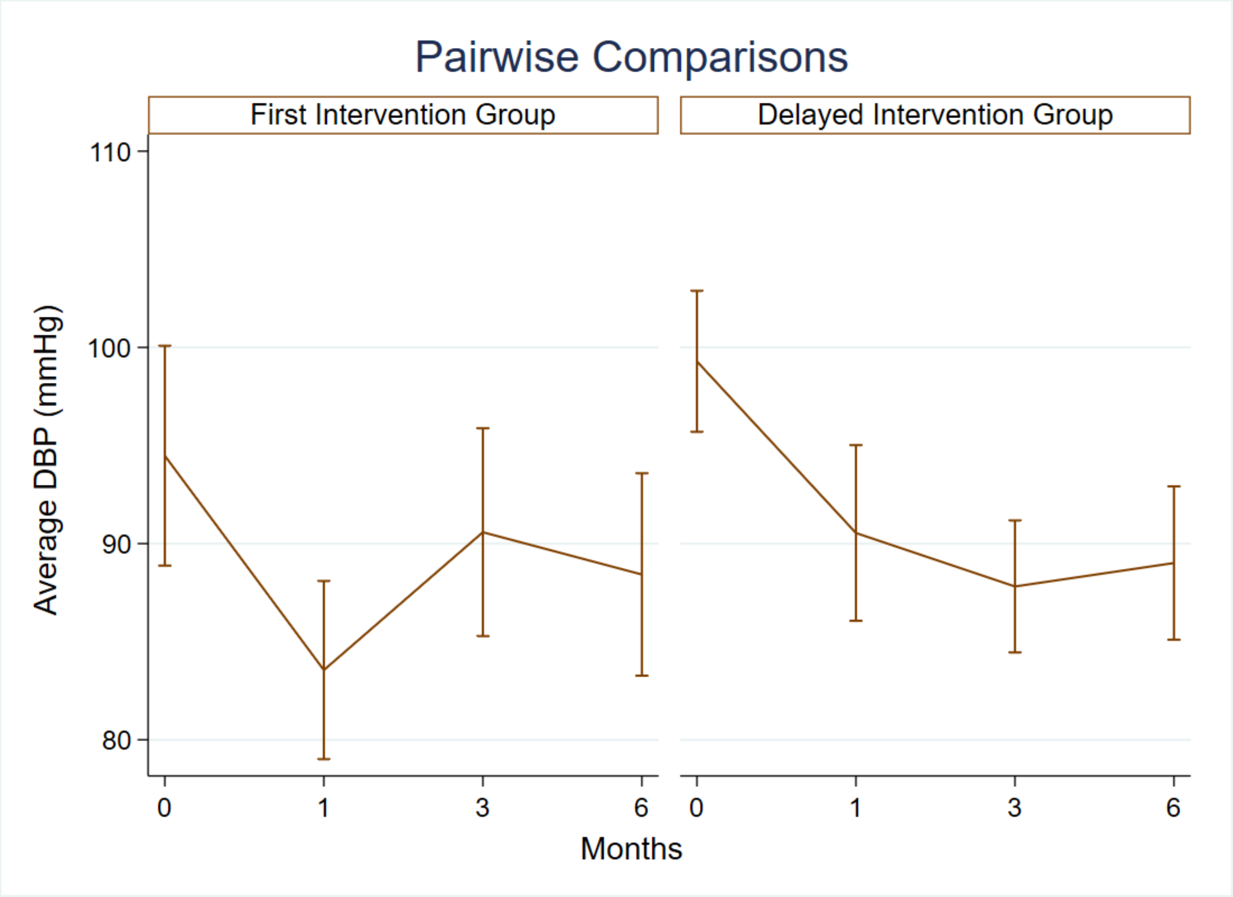

© 2025 Ogungbe O et al. *JAMA Network Open*.

eFigure 3. Model-Based Means for Hemoglobin A_{1c} by Study Group Over Time

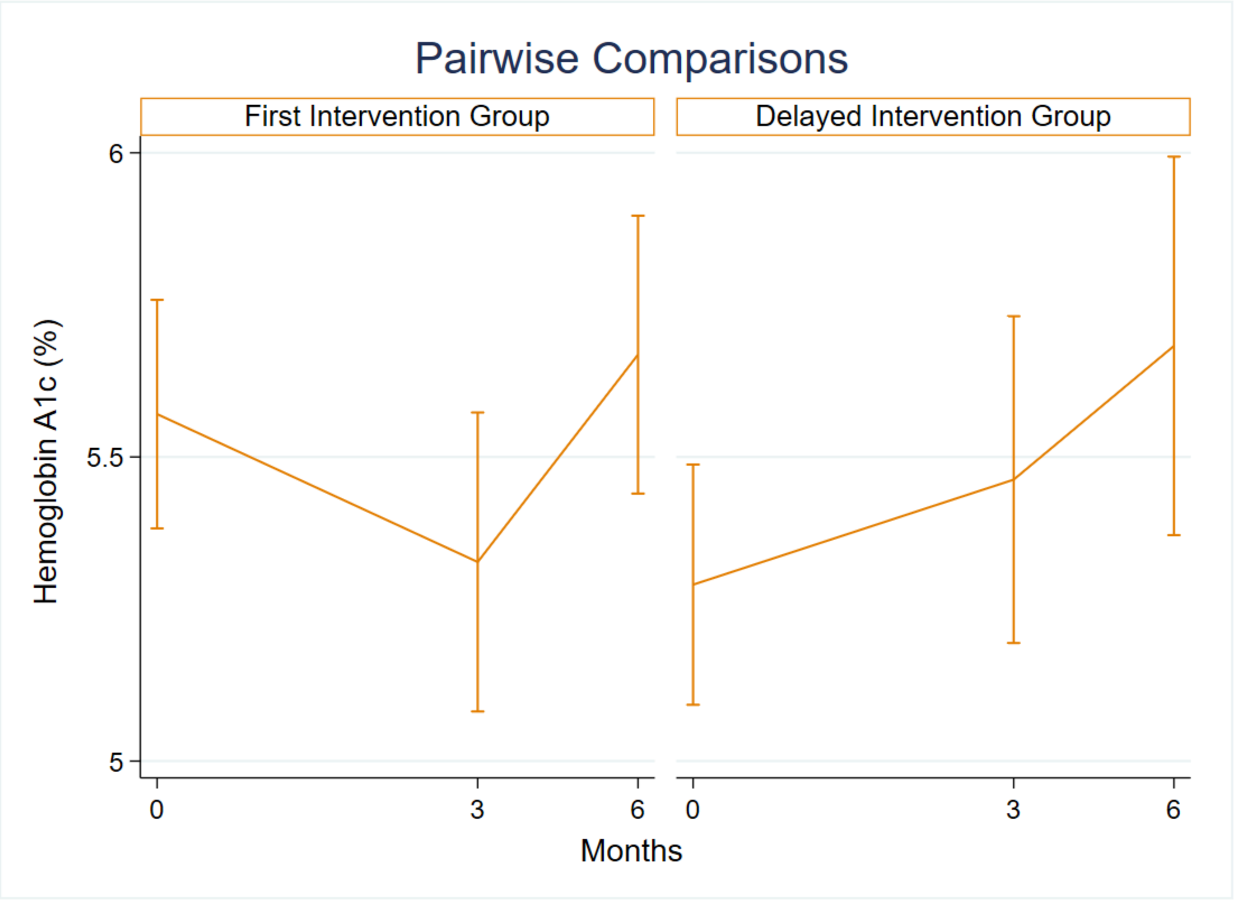

© 2025 Ogungbe O et al. *JAMA Network Open*.

eFigure 4. Model-Based Means for Body Weight by Study Group Over Time

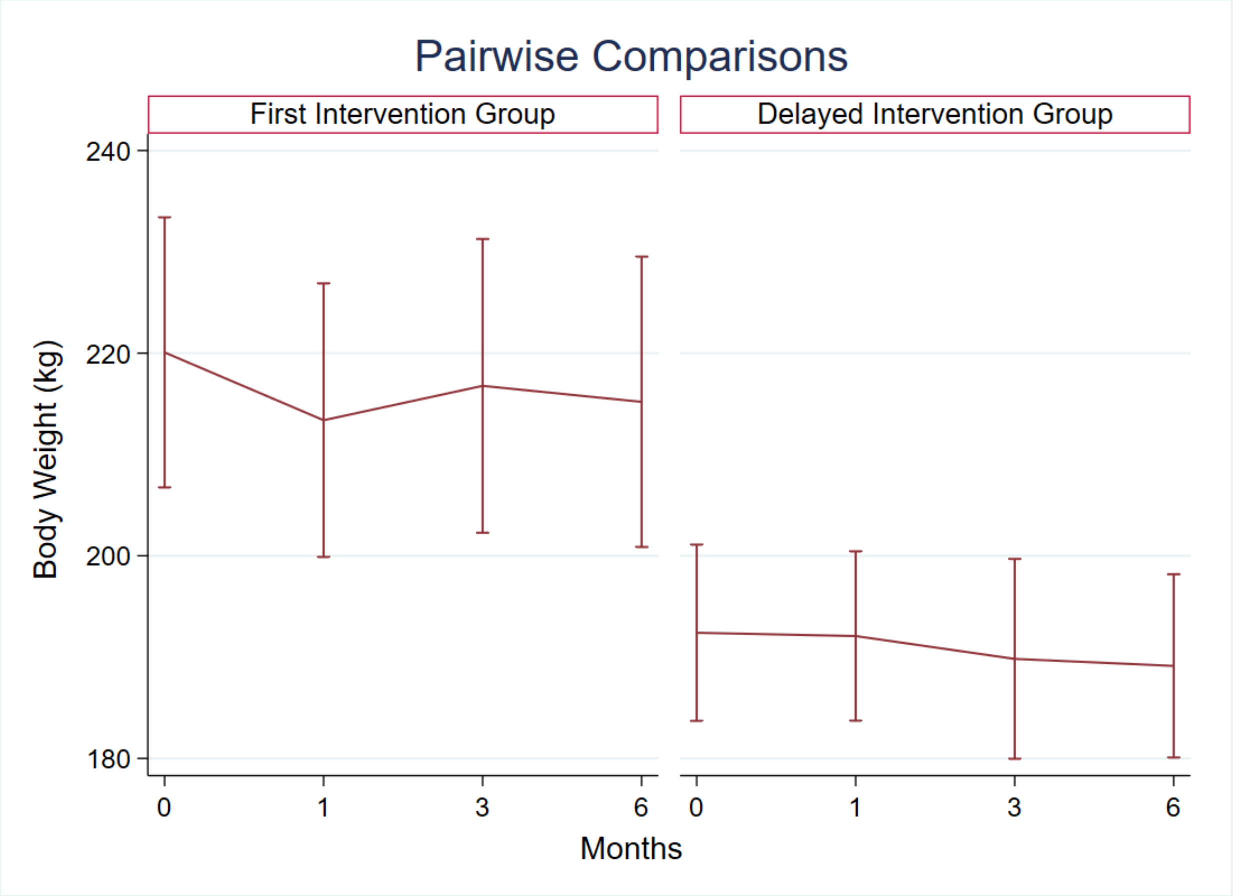

© 2025 Ogungbe O et al. *JAMA Network Open*.

eFigure 5. Model-Based Means for BMI by Study Group Over Time

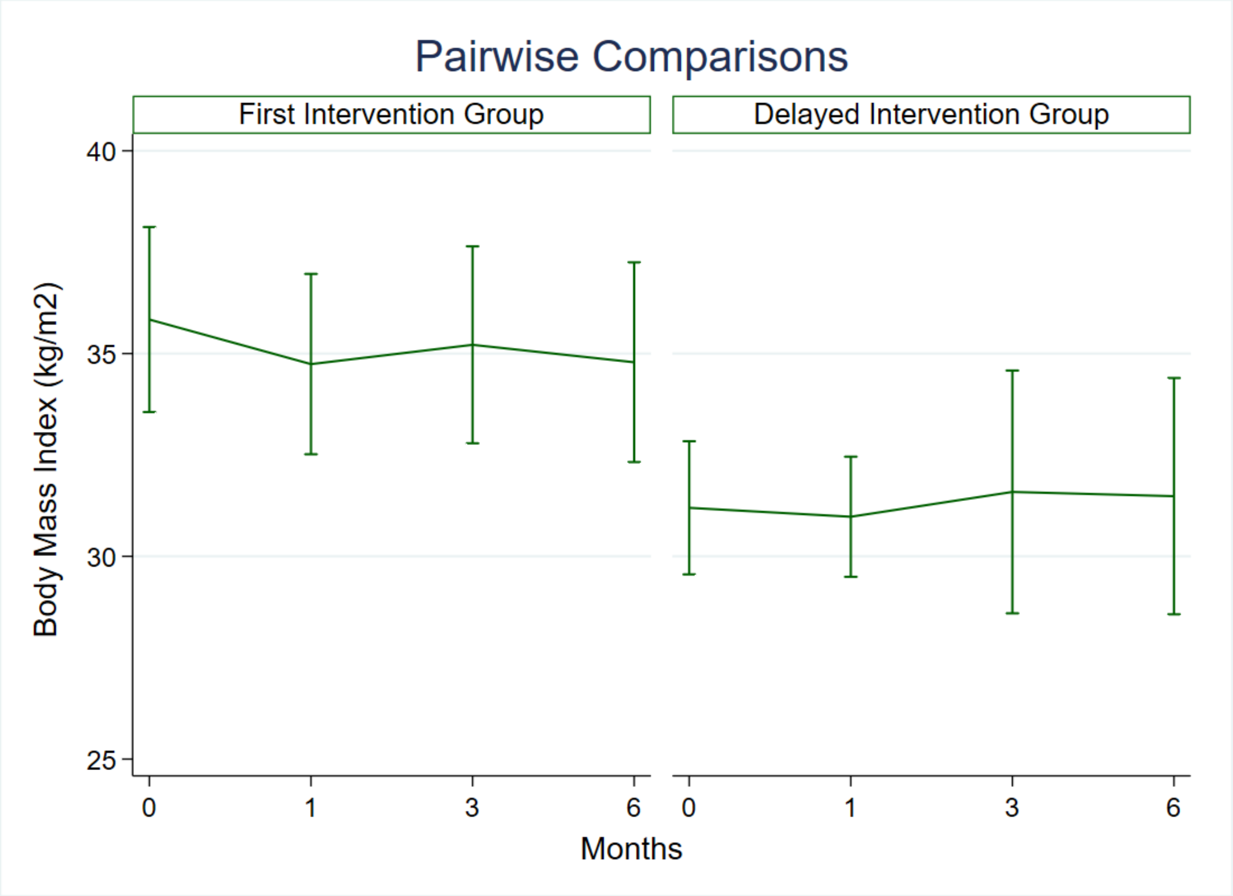

© 2025 Ogungbe O et al. *JAMA Network Open*.
