# Supplementary material for: A Virtual Cardiometabolic Health Program Among African Immigrants in the US: A Pilot Cluster-Randomized Clinical Trial
Source: JAMA Netw Open. 2025 Mar 4;8(3):e2462559. doi: 10.1001/jamanetworkopen.2024.62559 (PMC11880947; doi:10.1001/jamanetworkopen.2024.62559)
Supplement: Supplement 3. — Data Sharing Statement [file jamanetwopen-e2462559-s003.pdf]

Data Sharing Statement

Ogungbe. A Virtual Cardiometabolic Health Program Among African Immigrants in the US. *JAMA Netw Open*. Published March 04, 2025. doi:10.1001/jamanetworkopen.2024.62559

Data
Additional Information: ClincalTrials.gov Identifier NCT05144737
Data available: Yes
Data types: Deidentified participant data
How to access data: Data Request can be sent to: ycommod1@jhu.edu
When available: With publication

Supporting Documents
Document types: None

Additional Information
Who can access the data: Researchers whose proposed use of the data has been approved.
Types of analyses: Secondary data analyses or confirmatory analyses
Mechanisms of data availability: Approval of a proposal; Signed data access agreement
